# Prizes at the Académie De Médicine

**Published:** 1858-03

**Authors:** 


PRIZES AT THE ACADÉMIE DE MÉDECINE FOR 1857.—The Academy prize of 1000 francs, the Portal prize of 1000 francs, the Lefevre prize of 1800 francs, and the Barbier prize of 3000 francs have neither of them been adjudged this year, either in consequence of want of candidates, or of deficiency in merit in the essays offered. On the other hand, for the Argenteuil sexennial prize of 12,000 francs, for the greatest improvement in the treatment of stricture, during the period from 1850 to 1856, no less than 22 essays have been sent in, so that the committee is obliged to put off its report till next year. The Civrieux prize of 1500 francs, subject, "Nervous vertigo," has been adjudged to M. Max Simon; the first Capuron prize of 1000 francs, subject, "Sudden death in the puerperal state," has been adjudged to M. Mordret; and the second Capuron prize, also of 1000 francs, subject, "Saline mineral waters," has been adjudged to MM. E. Petrequin and Socquet, both professors at Lyons.

